# Braided Polyester Sutures with Different Manufacturing Characteristics in Cervical Cerclage: A Pilot Study Comparing Obstetric Outcomes and Operative Characteristics

**DOI:** 10.3390/jcm15145746

**Published:** 2026-07-22

**Authors:** Cem Terece, Simge Tezel Yozgat, Volkan Karataşlı

**Affiliations:** Department of Obstetrics and Gynecology, Balikesir Atatürk City Hospital, Balikesir 10100, Turkey; drsimgetezelyozgat@gmail.com (S.T.Y.); volkankaratasli@yahoo.com (V.K.)

**Keywords:** cervical cerclage, obstetric outcomes, operative characteristics, polyester suture, preterm birth, suture material

## Abstract

**Background and Objectives:** The effect of suture material on clinical outcomes in cervical cerclage remains debated. This pilot study compared two braided polyester suture materials with different manufacturing characteristics (Mersilene^®^ and Uniester^®^) in terms of obstetric outcomes and operative characteristics. **Methods:** Twenty-two pregnant women who underwent McDonald cerclage at a single center between January 2024 and December 2025 were retrospectively analyzed. Cases were divided into Mersilene^®^ (*n* = 11) and Uniester^®^ (*n* = 11) groups and compared regarding demographic characteristics, maternal complications, gestational age at delivery, neonatal outcomes, and operative time. Risk factors for preterm birth were assessed using multivariable logistic and linear regression analyses. **Results:** No significant differences were observed between groups in demographic, maternal, or neonatal outcomes. Median gestational age at delivery was 34.8 vs. 35.1 weeks (*p* = 1.00). Operative time was shorter in the Uniester^®^ group, although the difference did not reach statistical significance (21.1 vs. 24.4 min; mean difference 3.3 min, 95% CI: −1.5 to 8.1; *p* = 0.17). Multivariable analysis identified multiple pregnancy as the only independent predictor of preterm birth (OR = 12.5, 95% CI: 1.292–120.96, *p* = 0.029). **Conclusions:** The findings of this pilot study indicate that no statistically significant differences in obstetric or neonatal outcomes were observed between the two suture materials; given the limited sample size, this should not be interpreted as evidence of equivalence. The primary determinants of pregnancy prognosis were biological parameters, primarily multiple pregnancy, rather than suture material; preoperative cervical length showed only a non-significant trend toward association. The reportedly favorable handling characteristics and the non-significantly shorter operative time associated with Uniester^®^ may suggest a potential, though unconfirmed, technical advantage in terms of operative efficiency, particularly in time-sensitive or high-risk procedural contexts. Larger prospective multicenter studies are warranted to confirm these findings and to further evaluate whether differences in suture structure translate into clinically meaningful benefits.

## 1. Introduction

Cervical insufficiency is one of the leading causes of second-trimester pregnancy loss and preterm birth, characterized by painless cervical dilation and prolapse of the membranes into the cervical canal [1]. Preterm birth remains a major determinant of neonatal morbidity and mortality and is strongly associated with adverse outcomes such as respiratory distress syndrome, intraventricular hemorrhage, necrotizing enterocolitis, and long-term neurodevelopmental impairment [2,3]. Therefore, effective management of cervical insufficiency constitutes a critical component of modern obstetric practice.

Cervical cerclage remains the most commonly used intervention for prolonging pregnancy through mechanical cervical support in at-risk women. It is recommended for selected patients based on obstetric history, ultrasonographic findings, or physical examination [4]. Current guidelines and meta-analyses have demonstrated that cerclage reduces preterm birth rates and significantly prolongs gestational age when applied under appropriate indications [5,6,7].

Multiple factors influence the success of cervical cerclage, including the timing of the procedure, surgical technique (McDonald or Shirodkar), operator experience, and underlying obstetric risk factors. In addition, the characteristics of the suture material may also play a role in clinical outcomes [6,7,8,9]. Mersilene^®^ 5 mm polyester tape, a non-absorbable braided suture with a broad surface, is one of the most widely used materials in clinical practice due to its ability to distribute pressure evenly across the cervix [7,10]. However, alternative polyester-based cerclage materials produced by different manufacturers may differ in terms of weave density, surface roughness, thickness, and elasticity. These structural differences may potentially influence surgical handling and tissue response, as previously described for braided suture materials in general surgical contexts [11,12].

Previous studies in the literature have compared the thick 5 mm Mersilene^®^ tape with thinner polyester sutures or monofilament polypropylene sutures regarding pregnancy and neonatal outcomes; however, to our knowledge, no study has directly compared two polyester-based suture materials from different manufacturers [8,10]. Therefore, this study aimed to comparatively evaluate the effects of two polyester suture materials with different manufacturing and surface properties on obstetric outcomes and operative characteristics.

## 2. Materials and Methods

### 2.1. Study Design and Population

This study was designed as a pilot retrospective cohort study evaluating data from pregnant women who underwent cervical cerclage at the Department of Obstetrics and Gynecology, Balikesir Atatürk City Hospital, between January 2024 and December 2025. The study protocol was approved by the Scientific Research Ethics Committee of Balıkesir Atatürk City Hospital (approval code: 2026/03/45; approved on 26 March 2026) and conducted in accordance with the Declaration of Helsinki. All data were anonymized, and no patient-identifiable information was used. Patients with complete obstetric and neonatal outcome data were included in the analysis. Cases with missing follow-up data, those who underwent emergency (rescue) cerclage, and patients whose pregnancy outcome had not yet been ascertained at the time of data cutoff (*n* = 3; cerclage performed in November–December 2025, precluding completed follow-up) were excluded.

### 2.2. Group Allocation and Surgical Procedure

The suture material used for cerclage procedures was determined solely by hospital stock availability during the study period, as group allocation was not prospectively planned. Accordingly, patients were retrospectively categorized into either the Mersilene^®^ (Ethicon Inc., Somerville, NJ, USA) or Uniester^®^ (UNIMED—United Medical Industries Limited Co., L.L.C., Dubai Industrial City, Dubai, United Arab Emirates) group based on the suture used during the procedure. Uniester^®^ was predominantly used earlier in the study period, whereas Mersilene^®^ was used later once its stock was replenished; allocation was therefore sequential rather than randomized or fully contemporaneous. Toward the end of the study period, residual Mersilene^®^ stock was preferentially reserved for emergency (rescue) cerclage cases, which were excluded from the present analysis; consequently, elective cerclages during the transitional period were performed predominantly with Uniester^®^ until Mersilene^®^ procurement was replenished. In all cases, the standard McDonald cerclage technique was applied under spinal or general anesthesia according to patient preference and anesthesiologist assessment. With the patient in the lithotomy position, a purse-string suture was placed circumferentially around the cervix at the level of the internal os, incorporating four to six bites through the cervical stroma while avoiding the bladder and rectum, and the suture was tied anteriorly. All procedures were performed by a single maternal–fetal medicine specialist, and operative time was recorded as the interval between the first suture placement and completion of the final knot. Of note, the surgeon had greater prior clinical experience with Mersilene^®^ than with Uniester^®^, which was introduced more recently and used predominantly earlier in the study period.

### 2.3. Postoperative Management

According to the institutional protocol, all patients received a second-generation cephalosporin postoperatively in the absence of contraindications. To suppress uterine activity, indomethacin (25 mg every 6 h) was administered for the first 48 h. In addition, vaginal micronized progesterone (100 mg daily) was initiated postoperatively and continued until 37 weeks of gestation unless contraindications developed during follow-up.

### 2.4. Indications for Cerclage

Indications for cerclage were determined in accordance with the FIGO good practice recommendations for the prevention of preterm birth [7]. History-indicated (prophylactic) cerclage was offered to women with a history of one or more second-trimester pregnancy losses or spontaneous preterm births consistent with cervical insufficiency, and was performed electively between 12 and 14 weeks of gestation. Ultrasound-indicated cerclage was performed in women with a singleton pregnancy, a prior spontaneous preterm birth, and a transvaginal cervical length < 25 mm measured before 24 weeks of gestation. Since emergency cerclage cases were excluded, only these two indication categories (history-indicated and ultrasound-indicated) were included in the analysis.

### 2.5. Data Collection and Outcome Measures

Data were obtained by retrospectively reviewing the hospital automation system, electronic medical records, and patient files. Demographic variables (maternal age, gravidity, parity, BMI, smoking status), obstetric history (previous cerclage, multiple pregnancy), gestational age at cerclage, and preoperative cervical length were recorded. Gestational age at delivery, birth weight, and Apgar scores at 1 and 5 min were recorded. Maternal complications were defined as follows: PPROM was defined as rupture of membranes prior to 37 weeks of gestation before the onset of labor; chorioamnionitis was diagnosed clinically based on maternal fever (≥38 °C) accompanied by uterine tenderness, fetal or maternal tachycardia, or malodorous vaginal discharge; vaginal infection was diagnosed based on clinical examination and symptomatic discharge without routine culture confirmation; and early cerclage removal was defined as removal prior to the planned 37-week schedule due to labor, PPROM, infection, or bleeding. NICU admission and neonatal mortality (death within the first 28 days of life) were also evaluated as outcome variables. Length of NICU stay (days) was also recorded as a secondary neonatal outcome. In addition, the gestational week of cerclage removal—whether planned at term or early—was recorded for all patients.

### 2.6. Statistical Analysis

Statistical analyses were performed using IBM SPSS Statistics version 27.0 (IBM Corp., Armonk, NY, USA). Continuous variables were presented as mean ± standard deviation or median (minimum–maximum), while categorical variables were expressed as numbers and percentages. The normality of data distribution was assessed using the Shapiro–Wilk test. Group comparisons were conducted using the Student’s *t*-test or Mann–Whitney U test for continuous variables and Fisher’s exact test for categorical variables, as appropriate. Effect sizes were calculated alongside *p*-values for all group comparisons where they could be reliably estimated: mean differences with 95% confidence intervals (Welch’s approach) for normally distributed continuous variables, risk differences with 95% confidence intervals (normal approximation to the binomial) for categorical variables, and median differences with bootstrap-derived 95% confidence intervals (2.5th–97.5th percentile, 20,000 resamples) for non-normally distributed continuous variables. Confidence intervals were not calculated for three variables (cerclage removal week, interval from cerclage to delivery, and Apgar score at 1 min) due to distributional characteristics precluding reliable estimation from the available data. The primary outcome of this study was gestational age at delivery, analyzed as a continuous variable. Preterm birth before 34 weeks of gestation was defined as a key secondary outcome, analyzed as a binary variable to provide clinically interpretable risk estimates. To identify independent risk factors for preterm birth (<34 weeks of gestation), a multivariable logistic regression analysis was performed using clinically relevant variables selected a priori, based on prior literature, without a univariate pre-screening step. In addition, multivariable linear regression analysis was conducted to evaluate factors associated with gestational age at delivery. Covariates included in each multivariable model were selected based on clinical relevance, sample size constraints, and model parsimony, which resulted in slightly different variable sets between the logistic and linear regression models. A two-sided *p*-value < 0.05 was considered statistically significant.

### 2.7. Use of Generative AI

During the preparation of this manuscript, ChatGPT (GPT-4o, OpenAI, San Francisco, CA, USA) was used solely for language and grammar editing of the English text. The tool was not used for data analysis, interpretation of results, or generation of scientific content. All scientific content, analyses, and conclusions are the sole responsibility of the authors.

## 3. Results

### 3.1. Demographic and Clinical Characteristics

A total of 22 patients were included in the study, with 11 patients in each group (Mersilene^®^ and Uniester^®^). There were no statistically significant differences between the groups in terms of demographic or baseline clinical characteristics. The mean maternal age was 29.2 ± 4.4 years, and the mean BMI was 27.5 ± 3.2 kg/m^2^. The groups showed similar distributions with respect to gravidity, parity, smoking status, previous cerclage history, and multiple pregnancy rate (*p* > 0.05). No significant difference was observed between the groups regarding mean gestational age at cerclage (16.3 ± 3.5 weeks) or preoperative cervical length (21.1 ± 10.7 mm). Prophylactic cerclage was performed in 36.4% and ultrasound-indicated cerclage in 63.6% of patients in both groups, with no significant difference in cerclage indication between groups (*p* = 1.000). Detailed demographic and clinical data are presented in Table 1.

### 3.2. Maternal and Neonatal Outcomes

A comparison of maternal and neonatal outcomes according to suture material is presented in Table 2. The mean operative time was 3.3 min shorter in the Uniester^®^ group compared to the Mersilene^®^ group (24.4 ± 5.1 vs. 21.1 ± 5.7 min; mean difference 3.3 min, 95% CI: −1.5 to 8.1; *p* = 0.17), a difference that did not reach statistical significance but whose confidence interval does not exclude a clinically relevant effect. No statistically significant differences were observed between the groups in terms of maternal complications, including PPROM, chorioamnionitis, vaginal infection, bleeding, or early cerclage removal (all *p* > 0.05). The median gestational age at delivery was comparable between groups (34.8 weeks in the Mersilene^®^ group vs. 35.1 weeks in the Uniester^®^ group; median difference −0.3 weeks, 95% CI: −4.3 to 5.8; *p* = 1.00).

The rate of preterm birth before 34 weeks was similar in both groups (36.4%). Delivery rates before 32 weeks (27.3% vs. 18.2%, *p* = 1.00) and before 28 weeks (27.3% vs. 18.2%, *p* = 1.00) were also comparable between the Mersilene^®^ and Uniester^®^ groups. Neonatal outcomes, including birth weight (2360.7 ± 985.1 g), Apgar scores at 1 and 5 min, NICU admission rates, and neonatal mortality (9.1%), were also similar between groups (all *p* > 0.05). The median cerclage removal week was comparable between the Mersilene^®^ and Uniester^®^ groups (31.0 vs. 32.0 weeks, *p* = 0.88), as was the median interval from cerclage placement to delivery (9 vs. 10 weeks, *p* = 0.74). Although the median NICU stay was numerically longer in the Uniester^®^ group compared to the Mersilene^®^ group (35 vs. 4.5 days), this difference did not reach statistical significance (*p* = 0.13) and should be interpreted with caution given the small sample size and the wide range observed in both groups. Overall, none of the operative, maternal, or neonatal parameters assessed showed a statistically significant difference according to suture material.

The results of the multivariable logistic regression analysis are presented in Table 3. Multiple pregnancy was identified as the only independent predictor of preterm birth, significantly increasing the risk (OR = 12.5, 95% CI: 1.292–120.96, *p* = 0.029). Suture material type was not significantly associated with preterm birth. Although a 57% reduction in preterm birth risk was observed in the Uniester^®^ group, this did not reach statistical significance due to the wide confidence interval (OR = 0.429, 95% CI: 0.046–4.011, *p* = 0.458). Maternal age, BMI, and cervical length were not significantly associated with preterm birth risk.

The results of the multivariable linear regression analysis are shown in Table 4. Multiple pregnancy was the only independent factor significantly associated with a shorter gestational duration. Pregnancies with multiple gestations delivered, on average, 3.8 weeks earlier than singleton pregnancies (β = −3.842, *p* = 0.010). Cervical length demonstrated a non-significant trend toward prolonging gestation, with each 1 mm increase associated with an approximate delay of 0.5 days in delivery (β = 0.068, *p* = 0.099). Suture material type was not significantly associated with gestational age at delivery (β = 0.432, *p* = 0.663).

## 4. Discussion

The primary finding of this pilot study is that two different braided non-absorbable polyester suture materials used in cervical cerclage (Mersilene^®^ and Uniester^®^) demonstrated similar clinical efficacy in terms of gestational age at delivery and neonatal outcomes. Multivariable analyses revealed that suture material had neither an independent nor a significant effect on preterm birth risk or gestational age at delivery. Nevertheless, operative time was numerically shorter in the Uniester^®^ group, a finding examined in detail below in relation to material surface properties. Furthermore, multiple pregnancy was identified as an independent risk factor that significantly increased the risk of preterm birth (OR = 12.5, *p* = 0.029) and shortened gestational age by an average of 3.8 weeks (*p* = 0.010).

Previous studies have consistently demonstrated that cervical cerclage, when applied in appropriately selected patients, reduces the risk of preterm birth and prolongs pregnancy duration [1,4,13,14]. In the present study, the median gestational age at delivery (34–35 weeks) was consistent with previously reported outcomes, supporting the overall effectiveness of cerclage in this clinical context [13,14]; no statistically significant differences were observed between the two suture materials evaluated here.

The role of suture material in cerclage outcomes has been increasingly investigated; however, the available evidence remains inconsistent. For instance, Acar et al. [15] reported mean gestational ages at delivery of 34.4 and 37.9 weeks in the Mersilene^®^ and prolene groups, respectively, and found this difference to be statistically significant. Similarly, Deger et al. [8], in a study of 151 cases, reported delivery rates before 34 and 37 weeks of 57.3% and 80.5% in the Mersilene^®^ group, and 11.6% and 40.6% in the prolene group, respectively, concluding that obstetric outcomes were superior in patients who received prolene sutures. In contrast, a recent systematic review and meta-analysis including 2345 cases found no significant association between suture material and delivery before 37 weeks [16]. The inconsistency across the literature is likely attributable to heterogeneity among studies in terms of cerclage indications and patient selection. In the present study, two braided polyester sutures manufactured from the same base material but with different structural modifications and surface properties were compared, with no significant differences emerging across obstetric or neonatal outcomes—suggesting that surgical technique and appropriate patient selection may be more determinant of clinical outcomes than the specific type of suture material used.

**Figure 1:**
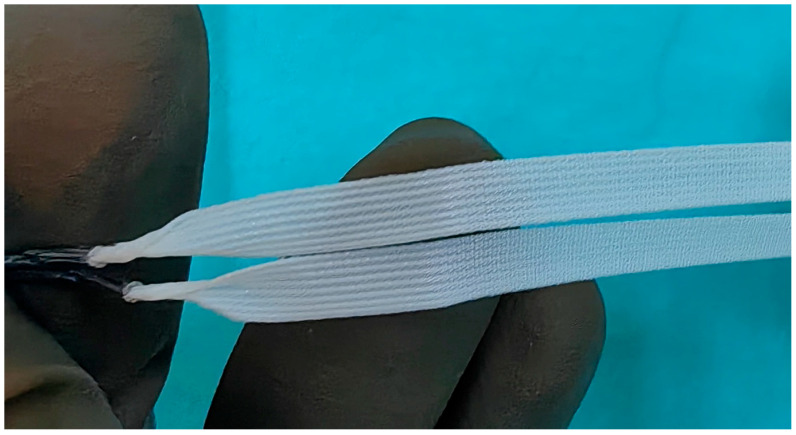
Mersilene^®^ braided polyester tape (5 mm). Note the dense braid pattern and relatively rougher surface texture.

**Figure 2:**
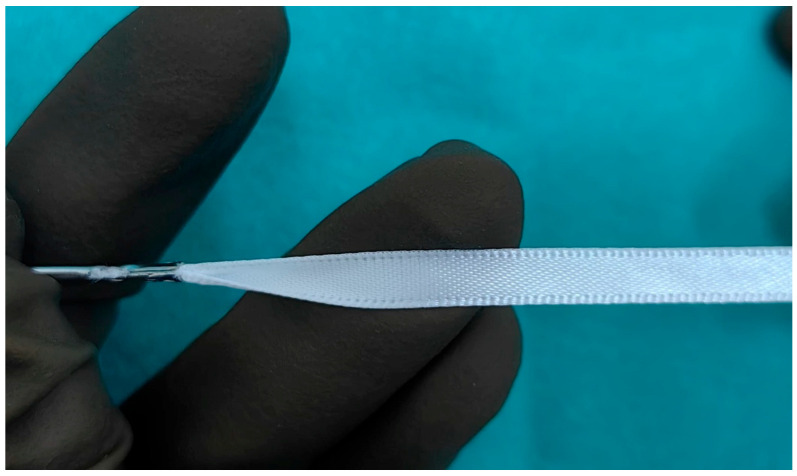
Uniester^®^ braided polyester suture. Note the smoother and more lustrous surface compared with Mersilene^®^.

The structural properties of braided suture materials, including braiding density and surface roughness, may influence tissue interaction and surgical handling. Macroscopic visual inspection by the surgical team, rather than standardized surface-roughness measurement, revealed that Mersilene^®^ has a denser braid and rougher surface, whereas Uniester^®^ appears smoother and more lustrous (Figure 1 and Figure 2). This qualitative observation should be interpreted as descriptive rather than quantitative. Although rougher surfaces have been associated with increased bacterial adhesion and biofilm formation [17,18], no differences were observed between the groups in terms of vaginal infection, PPROM, or chorioamnionitis in the present study. Nonetheless, the wide confidence interval for vaginal infection (risk difference −27.3%, 95% CI: −66.8% to 12.2%) indicates that this pilot study cannot exclude a potentially higher infection risk with Uniester^®^, and this possibility warrants attention in future adequately powered studies. This finding suggests that the primary determinants of postoperative infectious complications may be multifactorial, including the patient’s vaginal microbiota, surgical technique, perioperative antibiotic prophylaxis, and immune status, rather than the microstructural properties of the suture material. Similarly, Roman et al. [19] emphasized that complications following cerclage are influenced by multiple interacting factors.

Suture handling characteristics and knot security are recognized as subjective yet critical components of surgical success [11]. In our study, operative time was shorter in the Uniester^®^ group, although this difference did not reach statistical significance (21.1 vs. 24.4 min). We hypothesize that this finding may be related to differences in handling characteristics associated with the smoother surface of Uniester^®^, which could generate less “drag force” during tissue passage compared to Mersilene^®^; however, this mechanism was not directly measured in the present study and remains speculative. Studies on suture materials have shown that lower surface roughness not only facilitates surgical handling but also minimizes tissue trauma [12]. The improved handling characteristics of Uniester^®^ may provide a potential technical advantage, particularly in cases with fragile or edematous cervical tissue, in emergency or rescue cerclage procedures performed under time pressure, or when minimizing anesthesia exposure is clinically prioritized. Nonetheless, the observed difference in operative time (approximately 3.3 min) is modest in absolute terms; whether a difference of this magnitude constitutes a clinically meaningful benefit has not been established and would require larger studies with an a priori-defined minimal clinically important difference threshold, and this interpretation should therefore be regarded as hypothesis-generating rather than confirmatory.

Although the median NICU stay was numerically much longer in the Uniester^®^ group compared with the Mersilene^®^ group (35 vs. 4.5 days), this difference did not reach statistical significance (*p* = 0.13), and the following interpretation should be read with this limitation firmly in mind. This finding appears to run counter to the overall pattern of comparable outcomes between groups and is most plausibly explained by the small sample size and the resulting sensitivity of the median to a small number of infants with prolonged NICU admission, rather than by any suture-related mechanism; no plausible biological pathway links suture material to length of neonatal intensive care. Gestational age at delivery, birth weight, and neonatal mortality—arguably more direct indicators of neonatal status—were comparable between groups, further supporting the interpretation that this NICU-stay discrepancy reflects sampling variability in a small cohort rather than a true between-group difference. Larger studies are needed to confirm whether this observation persists or represents a chance finding.

Multivariable analyses identified multiple pregnancy as a strong and independent risk factor for preterm birth, with affected patients delivering approximately 3.8 weeks earlier compared to singleton pregnancies. This finding is consistent with previous studies emphasizing the significant role of multiple pregnancy as a risk factor for cervical insufficiency and preterm birth [20]. However, the efficacy of cervical cerclage in multiple pregnancies remains debated [20,21]. A Cochrane review published in 2014 reported insufficient evidence supporting the benefit of cerclage in multiple gestations and even suggested potential adverse outcomes in certain subgroups [21]. Although our pilot study was not sufficiently powered to evaluate the efficacy of cerclage specifically in multiple pregnancies, the findings indicate that the risk of preterm birth remains high in this patient group even after cerclage placement. Therefore, additional monitoring protocols and complementary intervention strategies may need to be considered in patients with multiple pregnancies.

Future research should aim to validate these preliminary findings through larger, prospective, and ideally randomized studies comparing polyester suture materials with different surface and manufacturing characteristics. Such studies would benefit from standardized, objective measures of surgical handling (e.g., instrumented drag-force testing) rather than subjective operator assessment, as well as microbiological evaluation of the vaginal microenvironment to clarify whether suture surface properties meaningfully influence infection risk. Multicenter designs would also allow adequately powered subgroup analyses in specific populations, such as multiple pregnancies, where the present pilot study suggests a persistently elevated risk of preterm birth despite cerclage placement.

## 5. Limitations

The main limitations of this study are as follows. Due to its pilot design, the sample size was limited, which may have prevented some of the favorable clinical trends observed in the Uniester^®^ group from reaching statistical significance (type II error). Furthermore, the limited number of outcome events relative to the number of covariates included in the logistic regression model may have resulted in overfitting and contributed to the wide confidence intervals observed (e.g., for multiple pregnancy); these estimates should therefore be interpreted with caution. As this pilot study evaluated gestational age at delivery as the primary outcome and preterm birth before 34 weeks as a secondary outcome without adjustment for multiple comparisons, findings from the secondary analysis should be regarded as hypothesis-generating rather than confirmatory. The fact that group allocation was based on hospital stock availability rather than randomization, following a sequential rather than fully contemporaneous pattern—with Uniester^®^ predominantly used earlier in the study period and Mersilene^®^ later, once stock was replenished—may have introduced uncontrolled confounding variables between the two groups, although the absence of significant between-group differences in any demographic, maternal, or neonatal parameter provides some reassurance against a major secular effect. Additionally, all procedures were performed by a single surgeon, which eliminates inter-operator variability as a confounder for the operative time comparison but limits the generalizability of the findings to other surgeons or centers; notably, the surgeon had greater prior familiarity with Mersilene^®^ than with the more recently introduced Uniester^®^, yet operative time was nonetheless shorter with Uniester^®^, arguing against a simple learning-curve explanation for this finding. Although operative time was recorded objectively, the assessment of surgical handling characteristics relied on the surgeon’s subjective experience rather than standardized measurement tools; therefore, these findings should be interpreted with caution. Similarly, suture surface roughness was assessed only by macroscopic inspection rather than quantitative measurement (e.g., profilometry), and vaginal infection diagnoses relied on clinical findings without culture-based or molecular microbiota analysis. Additionally, all patients received a standardized postoperative regimen (second-generation cephalosporin, indomethacin, and vaginal progesterone) regardless of suture material; while this protocol reflects real-world institutional practice, it may have attenuated any suture-related differences in infection risk or uterine irritability, potentially biasing the comparison toward the null. Due to its pilot design, no a priori sample size calculation was performed; the sample size was determined pragmatically by the number of eligible cases available during the study period, which likely limited the study’s ability to detect clinically relevant differences of modest magnitude.

## 6. Strengths

Despite its limitations, this study has several notable strengths. To the best of our knowledge, this is the first study to directly compare Mersilene^®^ and Uniester^®^ suture materials in cervical cerclage, which adds novelty to the existing literature. All procedures were performed in a single center using a standardized McDonald technique, and a uniform perioperative management protocol was applied to all patients. This methodological consistency minimizes procedural variability and reduces potential confounding related to surgical technique and postoperative care. In addition, the study provides a multidimensional evaluation by assessing not only obstetric and neonatal outcomes but also operative parameters such as operative time and handling characteristics. Finally, the study reflects real-world clinical practice, which enhances the external validity and practical applicability of the findings in routine obstetric care.

## 7. Conclusions

This pilot study found no statistically significant differences between two braided polyester suture materials (Mersilene^®^ and Uniester^®^) used in cervical cerclage in terms of maternal complications, gestational age at delivery, and neonatal outcomes; however, given the limited sample size and absence of an a priori power calculation, these findings should not be interpreted as demonstrating clinical equivalence. Although no statistically significant difference was observed in operative time between the groups, the relatively shorter operative time observed in the Uniester^®^ group may be related to its smoother surface characteristics and improved handling properties. Multivariable analyses indicate that pregnancy outcomes were primarily influenced by multiple pregnancy, whereas preoperative cervical length showed only a non-significant trend, rather than the type of suture material used. Given the limited sample size, these findings should be interpreted cautiously; however, they support that both materials can be used safely and effectively in clinical practice. Larger, prospective studies are needed to confirm these results and to further clarify potential differences in operative performance.

## Figures and Tables

**Table 1:** Demographic and clinical characteristics according to suture material.

Variables	Mersilene^®^ (*n* = 11)	Uniester^®^ (*n* = 11)	Total (*n* = 22)	*p*-Value
Maternal age (years)	29.8 ± 4.6	28.6 ± 4.2	29.2 ± 4.4	0.534
BMI (kg/m^2^)	27.9 ± 3.1	27.2 ± 3.4	27.5 ± 3.2	0.621
Gravidity, median (min–max)	3 (1–5)	2 (1–8)	2.5 (1–8)	0.468
Parity, median (min–max)	1 (0–3)	1 (0–2)	1 (0–3)	0.552
Smoking, *n* (%)	3 (27.3)	4 (36.4)	7 (31.8)	1.000 *
Previous cerclage, *n* (%)	3 (27.3)	3 (27.3)	6 (27.3)	1.000 *
Multiple pregnancy, *n* (%)	2 (18.2)	2 (18.2)	4 (18.2)	1.000 *
Gestational age at cerclage (weeks)	16.1 ± 3.4	16.5 ± 3.7	16.3 ± 3.5	0.795
Preoperative cervical length (mm)	21.4 ± 11.2	20.8 ± 10.8	21.1 ± 10.7	0.902
Indication, *n* (%)				1.000 *
Prophylactic	4 (36.4)	4 (36.4)	8 (36.4)	
Ultrasound-indicated	7 (63.6)	7 (63.6)	14 (63.6)	

Data are presented as mean ± SD, median (min–max), or *n* (%). * Fisher’s exact test; Student’s *t*-test or Mann–Whitney U test for continuous variables. BMI: body mass index.

**Table 2:** Comparison of maternal and neonatal outcomes according to suture material.

Variables	Mersilene^®^ (*n* = 11)	Uniester^®^ (*n* = 11)	Total (*n* = 22)	Effect Size (95% CI); *p*-Value
**Operative Characteristics**				
Operative time (min), mean ± SD	24.4 ± 5.1	21.1 ± 5.7	22.7 ± 5.5	MD 3.3 (−1.5, 8.1); 0.17 †
**Maternal Complications**				
PPROM, *n* (%)	2 (18.2)	2 (18.2)	4 (18.2)	RD 0.0% (−32.2, 32.2); 1.00 ‡
Chorioamnionitis, *n* (%)	2 (18.2)	1 (9.1)	3 (13.6)	RD 9.1% (−19.3, 37.5); 1.00 ‡
Vaginal infection, *n* (%)	5 (45.5)	8 (72.7)	13 (59.1)	RD −27.3% (−66.8, 12.2); 0.39 ‡
Bleeding, *n* (%)	2 (18.2)	3 (27.3)	5 (22.7)	RD −9.1% (−43.9, 25.7); 1.00 ‡
Early cerclage removal, *n* (%)	7 (63.6)	5 (45.5)	12 (54.5)	RD 18.2% (−22.7, 59.1); 0.67 ‡
**Pregnancy Course**				
Cerclage removal week, median (min–max)	31.0 (20.5–37)	32.0 (22–38)	31.5 (20.5–38)	0.88 †
Interval cerclage to delivery (weeks), median (min–max)	9 (1–20)	10 (1–22)	—	0.74 †
GA at delivery (weeks), median (min–max)	34.8 (20.5–37.8)	35.1 (22.0–37.3)	34.9 (20.5–37.8)	MD −0.3 wk (−4.3, 5.8); 1.00 †
Delivery < 34 weeks, *n* (%)	4 (36.4)	4 (36.4)	8 (36.4)	RD 0.0% (−40.2, 40.2); 1.00 ‡
Delivery < 32 weeks, *n* (%)	3 (27.3)	2 (18.2)	5 (22.7)	RD 9.1% (−25.7, 43.9); 1.00 ‡
Delivery < 28 weeks, *n* (%)	3 (27.3)	2 (18.2)	5 (22.7)	RD 9.1% (−25.7, 43.9); 1.00 ‡
**Neonatal Outcomes**				
Birth weight (g), mean ± SD	2447.7 ± 851.3	2273.6 ± 1103.9	2360.7 ± 985.1	MD 174.1 g (−706.3, 1054.5); 0.74 †
Apgar at 1 min, median (min–max)	7 (2–9)	7 (3–9)	7 (2–9)	0.65 †
Apgar at 5 min, median (min–max)	9 (5–10)	9 (6–10)	9 (5–10)	MD 0.0 (−1.0, 2.0); 0.72 †
NICU admission, *n* (%)	6 (54.5)	5 (45.5)	11 (50.0)	RD 9.1% (−32.5, 50.7); 1.00 ‡
NICU stay (days), median (min–max)	4.5 (1–25)	35 (3–84)	—	MD −30.5 days (−79.5, 19.0); 0.13 †
Neonatal mortality, *n* (%)	1 (9.1)	1 (9.1)	2 (9.1)	RD 0.0% (−24.0, 24.0); 1.00 ‡

Continuous variables: mean ± SD or median (min–max). † Mann–Whitney U test. ‡ Fisher’s exact test. GA: gestational age; NICU: neonatal intensive care unit; PPROM: preterm premature rupture of membranes. “—” indicates that the combined median for the total cohort is not presented, as medians cannot be directly derived from subgroup medians; group-level values (Mersilene^®^ and Uniester^®^) remain valid. MD: mean or median difference; RD: risk difference. 95% CIs for continuous variables were calculated using Welch’s *t*-test approach or bootstrap resampling (2.5th–97.5th percentile) for non-normally distributed variables; 95% CIs for categorical variables were calculated using the normal approximation to the binomial. CIs are not presented for cerclage removal week, interval to delivery, and Apgar at 1 min due to non-normal distributions precluding reliable estimation from summary statistics alone.

**Table 3:** Multivariable logistic regression analysis of risk factors for preterm birth (<34 weeks).

Variables	β	SE	Wald	*p*-Value	OR	95% CI
Suture material (Uniester^®^)	−0.847	1.142	0.551	0.458	0.429	0.046–4.011
Maternal age (years)	0.003	0.113	0.001	0.982	1.003	0.805–1.250
BMI (kg/m^2^)	0.105	0.125	0.710	0.399	1.111	0.870–1.419
Multiple pregnancy	2.525	1.159	4.742	0.029 *	12.500	1.292–120.96
Cervical length (mm)	−0.097	0.077	1.581	0.209	0.908	0.780–1.057

* *p* < 0.05. OR: odds ratio; CI: confidence interval; SE: standard error; BMI: body mass index. Model statistics: −2 Log Likelihood = 19.842, Nagelkerke R^2^ = 0.452, Hosmer–Lemeshow test *p* = 0.690.

**Table 4:** Multivariable linear regression analysis of factors associated with gestational age at delivery.

Variables	β	SE	t	*p*-Value	95% CI
Multiple pregnancy	−3.842	1.329	−2.891	0.010 *	−6.629 to −1.055
Cervical length (mm)	0.068	0.039	1.745	0.099	−0.014 to 0.150
Suture material (Uniester^®^)	0.432	0.975	0.443	0.663	−1.614 to 2.478
Constant	33.707	1.628	20.710	<0.001 *	30.294 to 37.120

* *p* < 0.05. β: regression coefficient; SE: standard error; CI: confidence interval. Model statistics: R^2^ = 0.391, adjusted R^2^ = 0.296, F(3,18) = 4.126, *p* = 0.022.

## Data Availability

The data presented in this study are available on request from the corresponding author. The data are not publicly available due to privacy restrictions.
